# Experiences of Family Caregivers of Older Patients with End-Stage Kidney Disease from Dialysis Initiation to End-of-Life Care: An Exploratory Qualitative Descriptive Study

**DOI:** 10.3390/nursrep16040108

**Published:** 2026-03-26

**Authors:** Natsumi Shimizu

**Affiliations:** Department of Nursing, Faculty of Nursing, Musashino University, 3-3-3 Ariake Koto-ku, Tokyo 135-8181, Japan; nshimizu@musashino-u.ac.jp; Tel.: +81-3-6865-1823

**Keywords:** nurse, advance care planning (ACP), end-stage kidney disease (ESKD), qualitative content analysis

## Abstract

**Background/Objective:** Older patients with end-stage renal disease who receive dialysis often discontinue treatment before the end of their lives. However, the trajectory of family caregiving in this specific context remains under-researched. This study explored the experiences of family members caring for older patients with end-stage kidney disease (ESKD), from the introduction of dialysis to end-of-life care. **Methods**: This qualitative descriptive study included three family members caring for older patients with end-stage renal disease who were undergoing dialysis in Japan. Data were collected through semi-structured, one-on-one interviews and analyzed using inductive qualitative content analysis within a qualitative descriptive design. **Results**: The results identified seven categories regarding the family’s experience from dialysis initiation to end-of-life care: Key findings, particularly regarding the terminal phase, included ‘shock of dialysis treatment discontinuation’, ‘last moments shared with the patient’, ‘nostalgic memories of the patient over time, and ‘reflections on end-of-life care for the patient.’ Families described a process wherein the sudden need for proxy decision-making, often without prior discussion, was linked to feelings of regret. **Conclusions**: The findings describe the continuous experiences of family caregivers in the Japanese context. These exploratory insights suggest that the absence of early Advance Care Planning may contribute to caregiver distress during the withdrawal phase. The results highlight the need for culturally sensitive renal supportive care that fosters communication and understanding of patients’ wishes to mitigate the ethical burdens on families.

## 1. Introduction

### 1.1. Background

End-stage kidney disease (ESKD) is a major global cause of morbidity and mortality, largely due to the aging population and the increasing prevalence of chronic kidney disease. Japan has one of the highest rates of initiation of renal replacement therapy in older patients with ESKD, and many patients receiving dialysis are older adults [1,2]. Consequently, Older patients with ESKD often face complex clinical trajectories that increase the burden of caregiving for their families. In this context, as patients age and comorbidities worsen, discontinuing dialysis in accordance with the patient’s wishes and quality of life has emerged as an important clinical and ethical issue [3]. Specifically, many older patients who initiate renal replacement therapy discontinue dialysis shortly before death [4,5], and most individuals die within two weeks of discontinuing dialysis [6].

However, discussions on end-of-life decisions, such as the withdrawal of dialysis and preferred place of death or prognosis, remain rare before this stage [7,8,9]. Additionally, when dialysis withdrawal is necessary, older patients often lack the cognitive ability or independence to make critical treatment decisions at the end-of-life [10]. Consequently, family members serve as surrogate decision-makers in the dialysis withdrawal process [11,12]. In Japan and other Asian societies, cultural norms often lead elderly individuals to entrust decisions to their families, making family involvement integral to the decision-making process [13,14].

Although these values can be supportive, they may also contribute to feelings of guilt and conflict for the family during end-of-life choices [15,16]. Previous studies have suggested that families frequently experience uncertainty regarding patients’ true intentions, psychological conflicts, and difficulties communicating with medical professionals [8]. Moreover, emotional burdens—including existential distress, demoralization, fear of death, and feelings of emotional unpreparedness—heavily affect family caregivers. Approximately one-third of caregivers experience high levels of existential distress, which can overwhelm their coping resources and complicate decision-making regarding end-of-life care choices [17]. Crucially, this psychological strain is often unrecognized and insufficiently addressed in usual care models, representing a significant gap in current support systems.

While previous studies highlight the prevalence of such distress, they often fail to capture the underlying mechanisms of why these decisions become emotionally overwhelming despite years of chronic care. Existing knowledge gaps remain regarding the specific emotional barriers and cognitive processes that families undergo when transitioning from “supporting life” to “ end-of-life and dying.”

Consequently, when making proxy decisions to discontinue dialysis without fully understanding the older patient’s wishes, families often face conflicts and challenges [18]. These conflicts, compounded by communication deficiencies and a lack of preparation, potentially lead to regret and complicated grief among surrogate family members [18]. Considering these factors, renal supportive care emphasizes the integration of comprehensive support tailored to address the psychological and social needs of patients and their families into conventional kidney replacement therapy-centered care, deeming the family’s perspective and dialogue with them as indispensable [19].

Previous family studies have primarily focused on perceptions of dialysis treatment, critical decision-making moments, and grief following bereavement [20]. While valuable, most of these studies have treated the “maintenance phase” and the “end-of-life phase” as separate entities. However, within the family unit, the experience is a continuous trajectory, starting with lifestyle changes at dialysis initiation, progressing through daily caregiving and witnessing physical decline, and culminating in end-of-life decisions. Quantitative scales cannot capture the nuances of how families’ perceptions of “care” transform over time, nor can cross-sectional qualitative studies fully reveal how the “daily routine” of dialysis influences the “crisis” of withdrawal.

These continuous experiences are believed to influence proxy decisions, subsequent regrets, and complicated grief. However, studies that comprehensively explore the qualitative transformation of family experiences remain limited. Therefore, to address this gap, a qualitative approach focusing on the trajectory of experiences is essential.

Understanding this continuous process is vital for identifying when and how to intervene to prevent traumatic decision-making. This study aimed to clarify the experiences of family caregivers of older patients with ESKD by tracing the qualitative transformation of their journey from the introduction of dialysis to end-of-life care.

### 1.2. Aim of the Study

This study aimed to qualitatively elucidate the series of processes experienced by the families of older patients with ESKD from dialysis initiation to end-of-life care. Understanding the overall structure of this process will provide key insights into predicting the difficulties experienced by families at each stage and developing consistent and effective nursing support from the decision-making preparation stage to post-bereavement care, thereby contributing to alleviating family suffering.

## 2. Materials and Methods

### 2.1. Design

This study adopted a descriptive qualitative design and performed inductive qualitative content analysis (QCA).

### 2.2. Participants

The study participants were family members of older patients who had received dialysis and for whom a proxy decision was made to discontinue treatment at the end of life. The inclusion criteria for family caregivers were as follows: (1) Families of deceased dialysis patients; (2) Individuals who served as the primary caregiver; and (3) Individuals who were directly involved in supporting the patient’s decision-making process. Exclusion criteria were: (1) Individuals with difficulty in verbal communication that would hinder the interview; and (2) Individuals for whom nurses at the cooperating hospitals determined that participation in a semi-structured interview would cause excessive psychological stress. Regarding the patients, ‘older patients receiving dialysis’ were defined as those who initiated dialysis at ≥65 years of age and had been on dialysis treatment for at least one year, regardless of modality. This one-year minimum duration was established to ensure that the caregivers had sufficiently experienced the continuous trajectory of care: overcoming the initial confusion of dialysis initiation, adapting to life with dialysis, and navigating physical decline toward the end of life.

Purposeful sampling was used to recruit participants. All study participants were recruited through the researcher’s network and hospitals willing to cooperate with the study. Nurses at the cooperating hospitals asked potential participants whether they were willing to listen to an explanation of the research. Recruitment was initiated by contacting over 20 hospitals and renal care facilities. However, most facilities declined assistance in contacting bereaved family members because of the perceived difficulties or ethical constraints associated with the sensitive nature of end-of-life decision-making and bereavement. Consequently, the final sample size was limited to three participants recruited through the researcher’s network and hospitals that agreed to cooperate with the study. Considering the exploratory nature of the study and the difficulty in recruiting this specific population (families who experienced dialysis withdrawal), a small but information-rich sample was selected.

### 2.3. Data Collection

Interviews were conducted between November and December 2023. To minimize the risk of psychological distress and respect the grieving process according to Japanese customs, we established an inclusion criterion that at least six months must have passed since the patient’s death.

This waiting period was established to ensure that the participants’ acute grief had settled, allowing for ethical data collection, while ensuring that their memories of the caregiving experience remained sufficiently clear. The interviews took place in a private conference room at the hospital where the patients had received end-of-life care. This setting was chosen to ensure strict privacy and a quiet environment physically separated from the ward where patients spent their final days, thereby creating a psychological distance from the immediate site of death. Interviews were conducted exclusively between the researcher and the participant, with no additional individuals present.

The author, who has experience in renal failure nursing and research, conducted the interviews. There was no prior relationship between the author and the participants. The interviews lasted between 60 and 67 min. All interviews were recorded in their entirety with the participants’ consent.

To mitigate potential social desirability bias arising from the institutional setting, the primary researcher explicitly informed all participants that this study was conducted independently of the clinical care team. It was guaranteed that the interview content would remain strictly confidential and that their participation—or lack thereof—would have no influence on their relationship with the hospital or the ongoing care of any other family members.

The interview guide was developed based on the research aim, relevant literature, and extensive clinical experience. To ensure content validity, the guide was reviewed and refined by an expert nurse specializing in bereavement care for patients with ESKD. Through this expert check, the appropriateness and sensitivity of the questions regarding the older patients’ situations at the beginning of dialysis treatment from the family’s perspective, life after starting dialysis treatment, older patients’ situations at the end of life, and proxy decision-making regarding dialysis treatment at the end of life were confirmed.

To manage potential emotional distress, the interviewer carefully monitored the participants’ condition throughout the session. If signs of distress appeared, the interviewer was prepared to pause to provide active listening and emotional support, prioritizing the participant’s well-being over data collection. Furthermore, a referral pathway was established to connect participants with mental health professionals in cases of severe grief or prolonged distress.

### 2.4. Data Analysis

Data were analyzed using inductive qualitative content analysis (QCA) [21]. This study examined the experiences of families caring for elderly patients with end-stage kidney disease, from the initiation of dialysis to the end-of-life care. These experiences involve the complex interplay of proxy decision-making by family members during the caregiving process and their emotions after the patient’s death. Given the fragmented nature of knowledge regarding this phenomenon, an inductive approach was deemed appropriate. The analysis was conducted in three phases: preparation, organizing, and reporting. First, the interviews were transcribed verbatim, and the researcher read the transcripts repeatedly to immerse themselves in the data. The unit of analysis was defined as a “meaning unit” (a constellation of words or sentences relating to the same central meaning) rather than a grammatical sentence, to preserve the context of the narratives. In the organizing phase, the abstraction process was performed through an iterative process of grouping and condensing. Initially, 749 open codes were generated. Through repeated comparison and sorting based on similarities and differences, these codes were gradually condensed to capture the underlying meanings. This process involved multiple levels of abstraction, moving from descriptive labels to conceptual categories. Ultimately, 70 codes were organized into 28 sub-categories and 20 generic categories, which were further synthesized into seven main categories representing the core structure of the families’ experiences. To ensure analytic transparency while protecting participant privacy, an example of the abstraction process is presented in Table 1. Regarding the structure of the results, although QCA typically organizes data by thematic similarity, the findings were presented chronologically. This structure was not arbitrarily imposed but reflects the natural “trajectory of caregiving” as narrated by the participants, from dialysis initiation to death.

### 2.5. Rigor and Trustworthiness

To ensure the trustworthiness of the research, the criteria for credibility, dependability, confirmability, and transferability were applied [21]. Positionality: The primary researcher has 5 years of clinical experience in nephrology nursing. This background facilitated rapport-building and a deep contextual understanding of the participants’ narratives regarding dialysis and end-of-life care. To mitigate potential bias arising from this expertise, the researcher maintained a reflexive journal throughout the study, consciously setting aside professional preconceptions to ensure findings remained grounded in the participants’ subjective experiences rather than clinical expectations. Credibility and Dependability: Credibility was established through rigorous peer debriefing sessions conducted bi-weekly over a four-month analysis period. Throughout the analysis, the primary researcher consulted with two nursing professors possessing extensive expertise in qualitative methodology to review all codes and sub-categories. This process of external audit ensured thematic consistency and rigorous abstraction. The primary researcher reflected on each phase of the analysis based on their feedback, ensuring the categorization process remained transparent and methodologically sound. Furthermore, to manage potential emotional distress, the interviewer carefully monitored the participants’ condition throughout the sessions. A referral pathway was established to connect participants with mental health professionals in cases of severe grief, although it was not required. The interview guide, developed based on the research aim and existing literature, was validated by an expert nurse in bereavement care to ensure its sensitivity. Transferability and Confirmability: Transferability was addressed by providing detailed descriptions of the participants’ context and care trajectory to allow readers to assess applicability. Member checking was not performed; given that the participants were bereaved family members, we determined that presenting them with a detailed analysis carried a significant risk of emotional re-traumatization. The reporting of this study followed the Consolidated Criteria for Reporting Qualitative Research (COREQ) checklist [22].

## 3. Results

### 3.1. Participant Demographics

Three women participated in this study. All the study participants lived with older patients, and two were employed. All three participants were the daughters of older patients (Table 2). The patients consisted of two mothers and one father, aged in their 70s or 80s at the time of death, and their dialysis treatment was hemodialysis. All patients were assessed as having intact cognitive function during the decision-making process regarding dialysis withdrawal. The average duration of the interviews was 60–67 min. The precise interval from dialysis withdrawal to death was not tracked in this study to prioritize the emotional safety of the bereaved family members and minimize their psychological burden during the interviews.

### 3.2. Family Experience of Older Patients with ESKD from Dialysis Initiation to End-of-Life Care

Overview of Findings: A total of 749 open codes were generated across the three participants (A: 234; B: 261; C: 254). Through an iterative process of comparison and condensation, these codes were organized into 28 sub-categories. These were further abstracted into 18 generic categories, and ultimately, seven main categories were identified representing the essence of the families’ experiences. Ultimately, seven main categories were identified: (1) benefits of dialysis treatment, (2) daily life with dialysis, (3) realization of the patient’s gradual decline based on physical changes, (4) shock of withdrawal of dialysis treatment, (5) last moments shared with the patient, (6) nostalgic memories of the patient over time, and (7) reflections on end-of-life care for the patient. Table 3 presents these seven main categories. To illustrate the content of these categories, representative subcategories are also presented. The following results show, in chronological order, how the events and emotional changes experienced by family members accumulated over time.

#### 3.2.1. Benefits of Dialysis Treatment

This category illustrates the family’s process of reappraising the value of the treatment. It links the initial acceptance of a limited prognosis with the eventual recognition that the treatment succeeded in balancing restrictions and longevity.

*For approximately a year, he went wherever he wanted to. It may have been difficult for him. However, he was able to do many things, and when I think of it, I feel that introducing dialysis was the right decision.*

(Participant A)

#### 3.2.2. Daily Life with Dialysis Treatment

This category highlights how families found positive meaning in their routines. It consists of two key aspects: maintaining normalcy in daily life despite medical constraints and finding joy through socialization within the dialysis setting.

*I looked forward to travelling with my family on days when I did not have dialysis treatment.*

(Participant B)

*I was able to meet people of that age group whom I would not have met otherwise, and I was able to communicate with them [other patients] in various ways, (I was able to meet them) because all of them were on dialysis.*

(Participant B)

#### 3.2.3. Realization of the Patient’s Gradual Decline Based on Physical Changes

This category captures the chronic process of observing physical deterioration during the maintenance phase of dialysis. Distinct from the acute shock of the withdrawal decision, this phase involves the family’s gradual recognition that the patient’s baseline health is fading, serving as a precursor to the end-of-life transition.

In this main category, family members reported that the older adult patient had exhibited a gradual decline in activities of daily living before death, even while undergoing dialysis treatment. Furthermore, the family members reported that before the older patient died, she could hardly eat and was visibly losing weight, which made them realize that her death was approaching slowly.

*More and more, I knew that she was getting weaker and weaker, you know.*

(Participant B)

*As she aged, her legs weakened. Whenever we went out, I would take her in a wheelchair because she had trouble walking.*

(Participant B)

#### 3.2.4. Shock of Withdrawal of Dialysis Treatment

Unlike the gradual realization of decline, this category represents the acute emotional impact of the specific decision to withdraw from treatment. It focuses on the psychological conflict and burden of proxy decision-making when families are forced to choose between life prolongation and the patient’s quality of life. This main category represents the family’s perspective when they must make a proxy decision to forgo dialysis. The family reported that the older patients chose to forgo dialysis because it was physically demanding for them. However, the family also reported that the older patient’s situation made them wonder whether they did not want to undergo dialysis and whether they would have been able to live their lives more freely without dialysis. The family understood that forgoing dialysis would result in the death of the older patient and reported that they could not think of anything else when making the decision.

*Even with dialysis, the same situation persisted. They said I could last for three, four, or five more days… However, I might not have done it because we felt that he was not going to get better anyway, even if we prolonged the tough situation.*

(Participant A)

*“Back then, whenever I brought the patient’s belongings to the ward, I was asked, ‘How is it going [regarding the decision to stop dialysis]?’ Different nurses came to me one after another to ask questions. It was like, ‘Wait, what’s the update on that?’ That really made me reluctant to visit the hospital.”*

(Participant C)

*(When we decided to stop dialysis, I cried behind the bed.) The nurse then rubbed my back. Even now, it is the toughest time for me.*

(Participant C)

#### 3.2.5. Last Moments Shared with the Patient

This category encompasses the enactment of the family’s final wishes following the withdrawal decision. It bridges the logistical success of realizing the wish to return home with the emotional importance of facilitating family gatherings before death.

*The nurse in charge was amazing, and I still feel indebted to her support. The nurse said that she really wanted her (my mother) to go home. Therefore, we ended up getting the decision approved by arranging for another nurse to accompany us [for the visit back home].*

(Participant C)

#### 3.2.6. Nostalgic Memories of the Patient over Time

This category focuses on the emotional recollection of the patient’s life and shared history. It involves maintaining a connection with the deceased through positive memories and symbolic objects as a mechanism for coping with loss. This main category reported that after the death of an older patient, the family members were in a state of shock for a while and could not look at items that reminded them of the patient or go to the dialysis room. After some time, once the family members had composed themselves, they reported discussing the memories of older patients with other family members using photographs.

*I do not remember the date, but it was sometime in mid-May. I thought to myself, ‘Oh, it’s been 7 years since the patient passed away.’ We were just talking about it the other day, and I was like, Oh, well, those days are gone. (I used to drive and take the patient for a drive.)*

(Participant A)

#### 3.2.7. Reflections on End-of-Life Care for the Patient

Unlike nostalgic memories, this category involves a cognitive evaluation of the care provided. This primary category included retrospective assessments of family decisions, encompassing feelings of regret, guilt, or justification regarding the place of death and the quality of end-of-life care. Families also realized only after the elderly patient’s death that they should have listened more to the patient’s thoughts and needs. The elderly patient prioritized the family’s wishes. However, family members reported hesitating to ask the patient about their views on life-sustaining treatment.

*(Hearing about another patient who was taken care of at home.) When she was no longer good at the end, I decided to take her home and care for her. I was slightly surprised to learn that this was possible.*

(Participant B)

*(Reflecting on the possibility of end-of-life care at home) That is how I felt. I had not considered this until now. I should have.*

(Participant C)

*“(When I received the form regarding preferences for life-prolonging treatment in the dialysis room,) the patient did not say much beyond that point. I also could not bring myself to ask or discuss how we should handle it, even once. I regret not being able to talk about it.”*

(Participant C)

## 4. Discussion

This exploratory study provides insights into the trajectory of the experiences of families of older patients with ESKD, from dialysis initiation to end-of-life care.

The process identified was characterized by a series of phases: the family’s adaptation to the patient’s life on dialysis, the shock of confronting the withdrawal of dialysis, and ultimately, feelings of regret during end-of-life care.

Initially, the families did not have a positive view of dialysis. However, as they continued to live with the treatment, they began to establish a ‘new normal’, with statements such as, ‘I look forward to family trips on days when I do not have dialysis. ‘However, this stability was disrupted abruptly. ‘Shock due to withdrawal of dialysis treatment’ arises when an older patient’s physical condition deteriorates to the point that dialysis can no longer be continued. The patient’s family understood that discontinuing dialysis treatment would mean that the older patient’s death was imminent; nevertheless, the burden of making this decision as a proxy was significant and challenging for the family.

These decisions encompass medical choices and the acceptance of patient deaths within the context of their daily lives that have sustained them. Even when recognized as unavoidable, such decisions can provoke feelings of helplessness and self-blame, aligning with the conflicts in proxy decision-making highlighted by Rogers et al. [16]. Furthermore, the pressure of decision-making affects the family dynamics. One participant reported difficulty visiting the hospital due to fear of being asked to make the withdrawal decision, supporting da Silva et al. ’s [23] finding that medical professionals’ communication influences decision-making. This underscores the unique nature of dialysis withdrawal decisions, which directly lead to death and significantly increase the psychological burden on families [9].

Following the decision, families experienced ‘Last moments with their patients.’ Participants described how they spent time with older patients in the hospital until their death and occasionally brought them home. These experiences allow families to fulfill their wish for their loved ones to spend their remaining time in peace. In addition, older patients could spend their final days after dialysis treatment in a place where they could enjoy psychological well-being [24]. In contrast, families of older patients wished to bring them home at the end of life and regretted being unable to choose the place of death. Importantly, this study suggests that family members’ regret may be associated not with the proxy decision to discontinue dialysis but with the lack of prior discussion. This highlights that the location of a patient’s final moments not only fulfills their wishes and ensures care until the end but may also influence the psychological well-being of their families afterwards. In addition, ‘reflections on end-of-life care’ became clear after the patient’s death.

Regarding the category of ‘Nostalgic memories of the patient over time’, while recalling the past is a common feature of general bereavement trajectories, it appeared to hold a specific function for these families. For years, their lives have been strictly regulated by the dialysis schedule and dietary restrictions. Recalling the “healthy past” allowed the families to reclaim the patient’s identity as a person, separating them from their identity as a “dialysis patient.” This process of “re-humanizing” the deceased may be particularly crucial in chronic disease contexts, where medical management dominates daily life.

Based on these identified processes, it appears that family members’ feelings of regret regarding older patients undergoing dialysis may be related not to the proxy decision to discontinue dialysis but to the perception of failing to discuss the patient’s preferred place of death within the family and consider the patient’s wishes about where they wanted to die. Previous studies have indicated that the concordance rate between patients’ end-of-life wishes and their family members’ understanding is often low [8]. Because families may not accurately perceive the patient’s true intentions without explicit discussion, the burden of proxy decision-making increases. Reflecting sentiments such as ‘We didn’t think that far ahead about life-sustaining treatment’ or ‘We should have listened more to the patient’s wishes,’ participants expressed a desire for earlier communication. Additionally, these regrets suggest that families may have been forced into proxy decision-making because of a lack of information, such as not knowing about the option of end-of-life care at home. This aligns with Davison’s observation that many patients desire information about end-of-life care [24,25,26].

These processes suggest that within the cultural context where end-of-life discussions are often avoided, the absence of dialogue during the stable ‘daily life with dialysis’ phase appeared to leave families unprepared. Consequently, the necessity of making proxy decisions under critical circumstances was perceived by families as a factor that amplified their subsequent regret. Thus, the ‘shock of discontinuing dialysis’ and ‘reflection on end-of-life care’ identified in this study can be interpreted as interlinked. Our findings generate the hypothesis that, particularly in Japanese care settings, insufficient dialogue may exacerbate the sense of crisis, which in turn colors the bereaved family’s retrospective appraisal of the care.

To mitigate such crises, honoring patients’ preferences is crucial, as highlighted in previous studies indicating that end-of-life discussions are associated with better caregiver bereavement adjustment [27]. Importantly, Silveira et al. [28] demonstrated that while a significant proportion of older adults lack decision-making capacity at the end of life, those with advance directives are far more likely to receive care consistent with their preferences than those without. This quantitative evidence supports our finding that gradual ACP initiation during the stable phase is essential. Such planning not only ensures that patients’ wishes are respected but also serves as a vital component of grief support for families by reducing the burden of uncertain proxy decision-making.

### 4.1. Limitations

The primary limitation of this study was the small sample size. As this was an exploratory qualitative descriptive study, data saturation was not assessed; instead, the focus was placed on the depth of individual narratives and “information power” rather than achieving theoretical saturation in the traditional sense. Consequently, the findings should be interpreted as a case series offering detailed insights into specific individual contexts rather than generalized results. Additionally, the sample was homogenous, consisting only of daughters, which limits the generalizability of the findings. However, a detailed explanation of the decision-making process offers theoretical transferability to similar cultural contexts where family-centered care is prevalent. Other limitations include potential selection bias; specifically, recruitment via professional networks may have favored families with more positive experiences or those more connected to medical professionals, potentially limiting the transferability of the findings to those with negative experiences. Finally, the study is subject to recall bias inherent to the retrospective design.

### 4.2. Implications for Clinical Practice

The findings suggest that the “shock” of dialysis withdrawal often stems from a gap between the family’s perception of “daily routine” and the actual “gradual physical decline” of the patient. Therefore, clinical support must extend beyond the final decision-making process. Specifically, dialysis nurses, who have frequent and long-term contact with patients, play a pivotal role in bridging this gap. They are uniquely positioned to assess frailty and cognitive changes during regular sessions and gently share these observations with families. By initiating “micro-conversations” about how these physical changes align with the patient’s values before a crisis occurs, nurses can help families gradually accept the changing trajectory. In contrast, the interprofessional team should facilitate the transition when the dialysis burden outweighs its benefits. Their role involves leading the shared decision-making process, providing symptom management, and offering ethical consultations to resolve family conflicts or guilt. Through this collaborative approach—where dialysis nurses support the “process of awareness” and the interprofessional team supports the “process of transition”—families can perceive proxy decision-making not as a crisis but as a logical transition collaboratively prepared with the patient. This approach preserves the patient’s dignity and significantly reduces the caregivers’ feelings of regret.

## 5. Conclusions

Although this study involved a limited number of participants, the in-depth analysis illuminated the complex emotional trajectory of family caregivers from the initiation of dialysis to the patient’s death. The “shock” of withdrawal highlights the critical need for continuous Advance Care Planning (ACP) that goes beyond simple documentation of preferences; it requires an ongoing dialogue that helps families bridge the gap between “living with dialysis” and “preparing for the end of life.” While further research with a larger and more diverse sample is needed to validate these patterns, this study provides a foundational framework for understanding the unique burdens of families in Japan’s aging dialysis population and underscores the necessity of supporting the family’s narrative construction long before the final decision is made.

## Figures and Tables

**Table 1:** Example of the data analysis process.

Meaning Unit	Code (70)	Sub-Category (28)	Generic Category (18)	Main Category (7)
*While the patient was hospitalized, he was unable to eat. I brought him high-calorie juice, but he barely consumed it, and I was at a loss as to what to do.* (Participant B)	Progressive loss of appetite and oral intake unresponsive to nutritional supplements	Progressive deterioration of physical function and nutritional status despite ongoing treatment	Realization of approaching death stemming from progressive physical deterioration	Realization of the patient’s gradual decline based on physical changes
*(On the day we decided to stop dialysis) It was right when we were discussing a temporary return home for the very next day.* (Participant C)	Suddenness of the withdrawal decision coinciding with plans for a temporary home visit	Mental freeze and shock caused by the abrupt decision to withdraw dialysis just before discharge	Acute psychological impact of the sudden treatment withdrawal	Shock of dialysis treatment withdrawal
*(When I expressed my desire to take him home to see relatives) The nurse told me, ‘If he goes home even for a short while, you can have him meet the relatives.’ So, I decided we should go home temporarily.* (Participant C)	The nurse’s coordination to ensure shared time between the patient and family during hospitalization	Facilitating shared family time and temporary discharge through tailored nursing support	Deep trust in medical professionals and flexible support tailored to individual situations	Last moments with the patient

**Table 2:** Participant demography.

Characteristic	Participant 1	Participant 2	Participant 3
Participant			
Relationship to patient	Daughter	Daughter	Daughter
Other household members (Lived with other family)	Husband, Grandchild, Grandchild’s wife, Great-grandchild	Father (Patient’s husband),	Father (Patient’s husband), Patient’s son
Living arrangement	Co-residing	Co-residing	Co-residing
Patient (Deceased)			
Age/Gender	80s/Male	80s/Female	70s/Female
ADL	Wheelchair user	Wheelchair user	Wheelchair user
Duration on dialysis	Approx. 1.5 years	Approx. 11 years	Approx. 1.5 years
Duration of caregiving	Approx. 1.5 years	Approx. 11 years	Approx. 1.5 years
End-of-Life Context			
Place of death	Hospital	Hospital	Hospital
Time from death to interview	Approx. 7 years	Approx. 2 years	Approx. 1 year

**Table 3:** The abstraction process: Linking representative subcategories to the seven main categories.

Main Categories (7)	Generic Categories (18)	Sub-Categories (28)
Benefits of dialysis	Ambivalent acceptance regarding the initiation of dialysis	Acceptance of inevitable dialysis initiation while harboring negative perceptions and regret
	Psychological adaptation to uncertainty through positive meaning-making	Acceptance of uncertain outcomes and finding solace in the patient’s longevity and fulfilled wishes
Daily life with dialysis treatment	Establishment of a new lifestyle incorporating dialysis and maintenance of enjoyment	Coexistence of restrictions for safety and permissive engagement, prioritizing the patient’s well-being
		Care system established through family support for daily living and service utilization upon initiation of outpatient dialysis
		Establishment of a ‘new normal’ integrating dialysis routine and family leisure
	Perceiving the patient’s consideration through their sincere character	Perception of the patient as a diligent and dignified family pillar
		Perceiving the patient’s gratitude and concern for the family through their interactions and remarks to others
Realization of the patient’s gradual decline based on physical changes	Realization of approaching death stemming from progressive physical deterioration	Visible and marked physical deterioration immediately prior to death
		Progressive deterioration of physical function and nutritional status despite ongoing treatment
		Recognition of inevitable death due to deterioration and lingering attachment to future life events
	Hope derived from temporary physiological improvements	Optimistic expectations for recovery based on temporary improvement and nutritional intake
	State of constant vigilance and anxiety regarding sudden deterioration	Fear of emergency notifications stemming from recurrent physical crises
Last moments shared with the patient	Deep trust in medical professionals and flexible support tailored to individual situations	Reaffirmation of the deep trust the patient placed in the physician through their response to sudden deterioration
		Facilitating shared family time and temporary discharge through tailored nursing support
	Sense of fulfillment derived from being able to stay by the patient’s side until the very last moment	Sense of fulfillment derived from being able to stay by the patient’s side until the very last moment
	Bridging feelings among relatives and acting as an intermediary to share disease awareness and coordinate care	Mediating between family hopes for continuation and the medical reality with empathy
		Consideration for other relatives’ conflicts and coordination to share the final moments
Nostalgic memories of the patient over time	Maintenance of a continuing bond after bereavement	Sustaining a spiritual connection with the deceased patient
	Lingering shock and a deep sense of loss immediately after bereavement, accompanied by reminiscence	Lingering shock and a deep sense of loss immediately after bereavement, accompanied by reminiscence
Reflections on end-of-life care for the patient	Unfulfilled feelings regarding the lack of proactive proposals from medical staff and lingering regrets regarding the end-of-life process	Unmet needs regarding hygiene care and proactive proposals tailored to the terminal phase
		Remorse over absence at death and reflection on the option of earlier withdrawal for home care
		Questioning the lack of consultation during COVID-19 isolation despite the patient’s usual reliance on nurses
	Emotional conflict arising from limitations in daily caregiving	Regret over inability to meet the patient’s requests amidst daily interactions
	Absence of communication between family and patient, and indirect confirmation of will via medical professionals	Patient’s decision-making capacity and expression of wishes to healthcare providers, which were unknown to the family
		Doubts regarding the patient’s true wishes and ambivalence toward life restrictions caused by continuing treatment
	Avoidance of end-of-life communication due to psychological barriers	Fear of discussing life-prolonging treatment and avoidance of confirming the patient’s wishes
Shock of withdrawal of dialysis treatment	Acute psychological impact of the sudden treatment withdrawal	Mental freeze and shock caused by the abrupt decision to withdraw dialysis just before discharge
	Ethical decision-making regarding dialysis withdrawal	Choosing to discontinue dialysis to prioritize relief from suffering over life prolongation in the face of medical futility

## Data Availability

The datasets presented in this article are not readily available because participants of this article did not agree for their data to be shared publicly. Requests to access the datasets should be directed to the corresponding author.

## Public Involvement Statement

No public involvement in any aspect of this research.

## Guidelines and Standards Statement

This manuscript was drafted against the COREQ (Consolidated Criteria for Reporting Qualitative Research) guidelines [22] for qualitative research.

## Abbreviations

The following abbreviations are used in this manuscript:

ESKD	End-Stage Kidney Disease
ACP	Advance Care Planning
QCA	Qualitative Content Analysis
